# Identification of Potential BRAF Inhibitor Joint Therapy Targets in PTC based on WGCAN and DCGA

**DOI:** 10.7150/jca.51551

**Published:** 2021-01-21

**Authors:** YaLi Han, XiaQing Yu, YuZhen Yin, Zhongwei Lv, ChengYou Jia, Yina Liao, Hongyan Sun, Tie Liu, Lele Cong, ZhaoLiang Fei, Da Fu, Xianling Cong, Shen Qu

**Affiliations:** 1 Shanghai Center for Thyroid Disease, Shanghai Tenth People's Hospital, Shanghai, China;; 2 Department of biobank, China-Japan Union Hospital of Jilin University, Changchun, Jilin Province, People's Republic of China;; 3 Department of Nuclear Medicine, Shanghai Tenth People's Hospital, School of Medicine, Tongji University, Shanghai, China;; 4 Department of Endocrinology and Metabolism, Shanghai Tenth People's Hospital, School of Medicine, Tongji University, Shanghai, China;; 5 Central Laboratory for Medical Research, Shanghai Tenth People's Hospital, Tongji University School of Medicine, Shanghai, China.

**Keywords:** papillary thyroid cancer, *BRAF*, biomarkers, targeted drugs

## Abstract

As the most common mutation in papillary thyroid cancer (PTC), B-type Raf kinase V600E mutation (*BRAF^V600E^*) has become an important target for the clinical treatment of PTC. However, the clinical application still faces the problem of resistance to BRAF inhibitors (BRAFi). Therefore, exploring BRAF^V600E^-associated prognostic factors to providing potential joint targets is important for combined targeted therapy with BRAFi. In this study, we combined transcript data and clinical information from 199 *BRAF* wild-type (*BRAF^WT^*) patients and 283 *BRAF^V600E^* mutant patients collected from The Cancer Genome Atlas (TCGA), and screened 455 BRAF^V600E^- associated genes through differential analysis and weighted gene co-expression network analysis. Based on these *BRAF^V600E^*-associated genes, we performed functional enrichment analysis and co-expression differential analysis and constructed a core co-expression network. Next, genes in the differential co-expression network were used to predict drugs for therapy in the crowd extracted expression of differential signatures (CREEDS) database, and the key genes were selected based on the hub co-expression network through survival analyses and receiver operating characteristic (ROC) curve analyses. Finally, we obtained eight BRAF^V600*E*^-associated biomarkers with both prognostic and diagnostic values as potential BRAFi joint targets, including *FN1*, *MET*, *SLC34A2*, *NGEF*, *TBC1D2*, *PLCD3*, *PROS1*, and *NECTIN4*. Among these genes, FN1, MET, PROS1, and *TBC1D2* were validated through GEO database. Two novel biomarkers, PROS1 and *TBC1D2*, were further validated by qRT-PCR experiment. Besides, we obtained four potential targeted drugs that could be used in combination with BRAFi to treat PTC, including MET inhibitor, ERBB3 inhibitor, anti-NaPi2b antibody-drug conjugate, and carboplatin through literature review. The study provided potential drug targets for combination therapy with BRAFi for PTC to overcome the drug resistance for BRAFi.

## Introduction

As the most common subtype of thyroid cancer, papillary thyroid cancer (PTC) accounts for more than 85% of thyroid cancers^1^, ^2^. Though PTC is an indolent disease with a good prognosis, its incidence is increasing with years^3^. B-type Raf kinase *^V600E^* mutation (*BRAF^V600E^*) is the most common point mutation closely related to the recurrence of thyroid cancer and PTC-specific death^4^-^6^. Numerous studies on the carcinogenic molecular mechanisms of *BRAF^V600E^* have shown that *BRAF^V600E^* is related to the malignant progress of thyroid cancer^7^. Several BRAF inhibitors (BRAFi) for thyroid cancer have been approved by food and drug administration in America, such as vemurafenib, dabrafenib, etc^8^. However, a major challenge in clinical practice is the drug resistance to BRAFi^9^, ^10^. Therefore, exploring *BRAF^V600E^*-associated prognostic factors to providing potential joint targets is important for combined targeted therapy with BRAFi.

Previous studies have identified *BRAF^V600E^*-associated genes with diagnostic value for PTC based on weighted gene co-expression network analysis (WGCNA), which clusters genes into modules through calculating the expression correlations and topological overlaps between genes^11^, ^12^. However, the prognostic factors-associated with *BRAF^V600E^* based on large-scale clinical data need to be further investigated. Another study has explored the function of PTC-associated genes with both WGCNA and differential gene correlation analysis(DGCA), which measures the gene co-expression variations based on differential correlation calculations^13^, ^14^. Their study also predicted meaningful drugs for therapy through the CREEDS database, a database including single gene perturbations, single drug perturbations, and disease signatures from Gene Expression Omnibus (GEO) data^15^. However, the study didn't reveal any potential prognostic factors or drug targets for PTC, especially those related to BRAF mutations. To sum up, the current studies haven't explored potential prognostic biomarkers and drugs targeting genes associated with *BRAF^V600E^*.

In this study, we constructed a *BRAF^V600E^*-associated co-expression network and predicted meaningful drugs in CREEDS through combing WGCNA with DCGA for the first time, providing further insight into the regulatory mechanism of *BRAF^V600E^* mutation for PTC. Based on the co-expression network, we revealed eight *BRAF^V600E^*-associated key genes with both prognostic and diagnostic values for *BRAF^V600E^* PTC. Of note, our study revealed 2 novel BRAF^V600E^-related biomarkers have potential associations with the recurrence of PTC through a combination of database analysis and qRT-PCR experiment. This provided possible treatment strategies and potential drug targets for combination therapy with BRAFi for PTC to overcome the drug resistance for BRAFi.

## Material and Methods

### Data retrieval

Varsan2 somatic mutation data of 487 PTC patients was download by R package “TCGAbiolinks”^16^. The counts data of RNA high throughput sequencing (HTSeq-counts) of 502 PTC patients, including 58 normal tissues and 502 tumor tissues, was downloaded by R package “GDCRNAtools” from TCGA^17^. The low-expression gene was defined as the gene with the value of count-per-million (CPM) < 2 in more than half of the samples. After filtering out low expression genes, 13483 protein-coding genes (messenger RNA, mRNA) were extracted by transformed with GENCODE (https://www.gencodegenes.org/). Clinical information of the 502 patients was downloaded from UCSC Xena by R package “UCSCXenaTools”^18^. By intersecting 502 PTC patients with expression data and 487 PTC patients with mutation data, 482 cases meeting the following criteria were included in our analysis: Patients with clear BRAF mutation information; Patients with gene expression data of RNA-sequencing. Raw data of 108 PTC samples with specific *BRAF* mutation status were collected from three microarray datasets (accession numbers: GSE27155, GSE54958, and GSE58545) in the GEO database (https://www.ncbi.nlm.nih.gov/geo/). The expression data were processed as the previous description^11^.

### Ethical statement

Since the data involved in humans were all publicly available in the GEO dataset and TCGA dataset, the ethics committee approvals were stated in the primary studies and there was no additional ethical statement to be declared. The approval of data utilization of GEO dataset can be retrieved from https://www.ncbi.nlm.nih.gov/geo/info/disclaimer.html, and the approval of data utilization of the TCGA dataset can be retrieved from https://www.cancer.gov/about-nci/organization/ccg/research/structural-genomics/tcga. The human specimens used for protocol employed were approved by the Ethical Committee of China-Japan Union Hospital of Jilin University. The experimental procedures have been carried out by following the ethical standards as of the Declaration of Helsinki. Informed consent was obtained from all individual participants included in the study.

### Differentially expressed genes (DEGs) filtering

According to the mutation information of the *BRAF* gene showed in** table 1**, tumor samples of PTC patients were divided into *BRAF^V600E^* group (283 cases) and *BRAF^WT^* group (199 cases, including one case with *BRAF* nonsense mutation, 3 cases with other *BRAF* mutation type). DEGs between the two groups (WT-V600E DEGs) were screened by the “DESeq2” R package^19^. The *p*-value < 0.05, and the absolute value of log2(fold change) (*BRAF^V600E^* group vs. *BRAF^WT^* group) >1 were considered as the threshold of meeting significant difference. DEGs between normal tissues and tumor tissues were screened with the same method and was defined as N-T DEGs. DEGs specific to *BRAF^V600E^* PTCs were obtained by taking an intersection between WT-V600E DEGs and N-T DEGs. The matrix of mRNA counts was normalized by the “DESeq2” package based on variance stabilizing transformations.

### WGCNA

To explore genes related to the clinical traits we are interested in, including TMN stage, gender, age, mutation status of *BRAF*, WGCNA was conducted on the expression profiling of 3371 genes, which was the top 25% highest variance across 482 samples with full clinic information. An unsigned network was constructed with the soft threshold power = 6 (R^2^ = 0.899, slope = -1.348), which was calculated using nearly scale-free topology. Modules with > 30 genes were generated. The minimum cut height was set to 0.25 based on the dynamic tree cut algorithm. The correlation coefficients between the interested clinical traits and these modules were calculated with R package “WGCNA”. The *p*-value of less than 0.01 was defined as statistically significant. Module eigengene was defined as the first principal component of the expression matrix of the corresponding module. For each module, the correlation coefficient between the module eigengene and the gene expression profile was defined as module membership (MM). Gene Significance (GS) was characterized by the correlation coefficient between the individual gene and the clinical factors. DEGs specific to *BRAF^V600E^* PTCs in the module most positively correlated with *BRAF^V600E^* mutation were screened as *BRAF^V600E^*-associated genes. Among these genes, hub genes, of which the absolute values of both MM and GS ranked in the top 50, were selected for constructing the co-expression network.

### Function, pathways, and disease enrichment analysis

Function, pathway, disease enrichment analyses were performed on *BRAF^V600E^*-associated genes with R package “GDCRNAtools”, including Gene Ontology (GO) enrichment analysis, Kyoto Encyclopedia of Genes and Genomes (KEGG) pathway enrichment analysis, and Disease Ontology (DO) enrichment analysis based on GO (http://geneontology.org/), KEGG (https://www.genome.jp/kegg/) and DO (https://disease-ontology.org/) database respectively. GO supports many species with GO annotation query online via AnnotationHub, and KEGG Pathway and Module with the latest online data supports more than 4000 species listed in http://www.genome.jp/kegg/catalog/orglist.html. The false discovery rate (FDR) < 0.05 was considered statistically significant. The minimum size of the gene sets is set as 5.

### Differential correlation analysis

Differential correlation analysis between *BRAF^V600E^* PTC and *BRAF^WT^* PTC was conducted based on *BRAF^V600E^*-associated genes with R package “DGCA”. The absolute value of the correlation coefficients greater than 0.3 and p-value<0.05 were defined as significant gene-pairs. When the regulation of the two genes was opposite in the *BRAF^V600E^* and the *BRAF^WT^* group, the gene pair was considered to have a differential correlation between the two groups. Finally, the *BRAF^V600E^*-associated co-expression network based on the correlations between the hub genes was constructed and visualized by Cytoscape 3.7.2.

### Targeted drug prediction

Targeted drugs of the *BRAF^V600E^***-**associated genes with differential correlation were predicted with the opposite signatures from original crowdsourcing signatures in the CREEDS database(http://amp.pharm.mssm.edu/creeds/). The ranking of the predicted drugs was based on the Signed Jaccard Index, which represents signatures of reverse effects.

### Survival analysis

Relapse survival analyses of the hub genes were conducted with the “survival” and the “survminer” R package based on Kaplan-Meier mothed. The minimal proportion of observations per group was set to 0.25. Log-rank *p*-value < 0.05 was considered as significant statistically.

Expression differences of prognosis**-**associated genes between *BRAF^V600E^* and *BRAF^WT^* PTCs were validated in 108 patients collected from GEO using the nonparametric Wilcoxon rank-sum test. *p-*value < 0.05 was considered statistically significant.

### Receiver operating characteristic curve (ROC) analysis

Using SPSS 22.0 and R package “pROC”, ROC analyses of the prognostic indicators were conducted on patients from TCGA and GEO respectively. Genes with area under curve (AUC) > 0.90 and *p*-value < 0.05 were considered to have significant diagnostic value.

### RNA Extraction and qPCR

Tissue samples from 92 PTC patients were used to extract total RNA as the previous description^20^. Among the 92 PTC cases, 83 cases had paired normal and tumor tissue samples and 82 cases had clear *BRAF* mutation information, including 56 *BRAF^V600E^* PTC patients and 26 *BRAF^WT^* patients. The *BRAF* mutation status of these PTC patients was detected through Sanger sequencing in Sangon Biotech company with both forward and reverse primers in **table 2**. Total RNAs were reversely transcribed into cDNAs with PrimeScript^™^ RT reagent Kit (TAKARA, RR037A). Quantitative real-time PCR (qRT-PCR) analysis was performed on each cDNA template with 2×SYBR Green qPCR Master mix (Bimake, B21203) through Bio-Rad CFX96 Real-Time system. GAPDH was set as an internal control for gene quantification. The differences between groups for *in vitro* studies were analyzed by nonparametric t-test in GraphPad, with statistical significance (*p* < 0.05).

## Results

### DEGs screening

**Figure 1:**
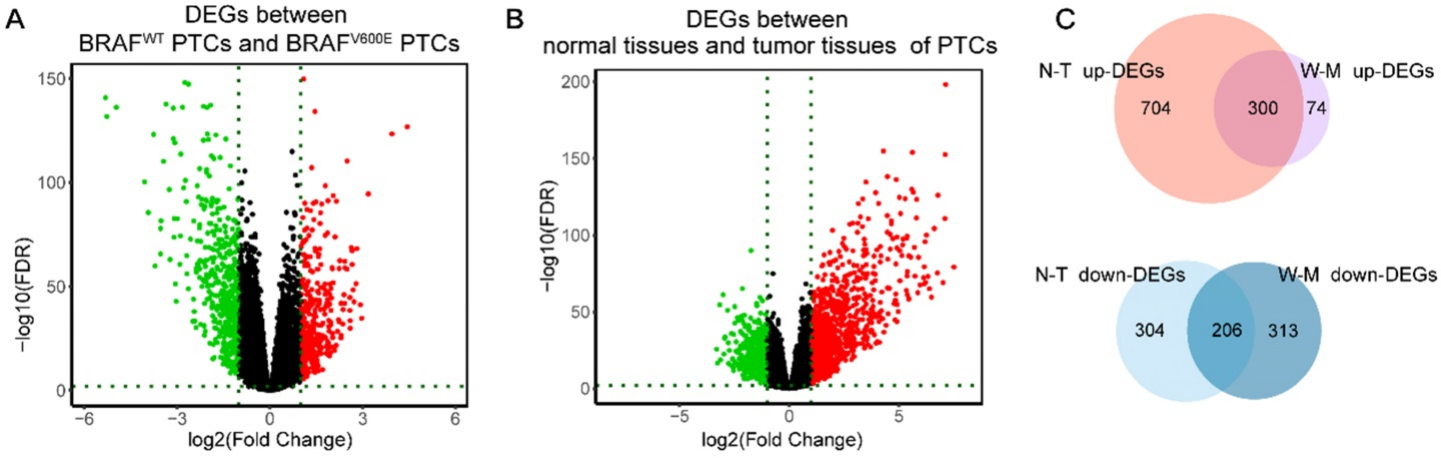
**Identification of DEGs. (A)** Volcano maps of aberrantly expressed mRNAs between two groups: *BRAF^V600E^* PTCs and *BRAF^WT^* PTCs. Red dots represent up-regulated genes and green dots represent down-regulated genes. **(B)** Volcano maps of aberrantly expressed mRNAs between two groups: normal PTCs and tumor PTCs. Red dots represent up-regulated genes and green dots represent down-regulated genes. **(C)** Venn diagram showed the intersection of DEGs between *BRAF^V600E^* PTCs and *BRAF^WT^* PTCs and DEGs between normal PTCs and tumor PTCs. DEGs: differential expression genes; FDR: False Discovery Rate; N-T up-DEGs: up-regulated DEGs between normal and tumor PTC tissues; N-T down-DEGs: down-regulated DEGs between normal and tumor PTC tissues; W-M up-DEGs: up-regulated DEGs between *BRAF^V600E^* and *BRAF^WT^* PTC tissues; W-M down-DEGs: down-regulated DEGs between *BRAF^V600E^* and *BRAF^WT^* PTC tissues.

Firstly, we analyzed the difference in gene expression between *BRAF^WT^* and *BRAF^V600E^* groups. A total of 893 DEGs were obtained, among which the expression of 374 genes was up-regulated, and the expression of 519 genes was down-regulated (**figure 1A**). Next, we got 1514 DEGs in the comparison between normal and tumor tissues, including 1004 highly-expressed genes and 510 under-expressed genes (**figure 1B**). Finally, by intersecting the up-regulated genes and down-regulated genes of the two differential analyses, we obtained 506 DEGs specific to *BRAF^V600E^* PTC, of which 300 were up-regulated and 206 were down-regulated (**figure 1C**). The remained 1008 DEGs between normal tissues and tumor tissues were defined as non-*BRAF^V600E^*-specific DEGs.

### Turquoise modules are the key *BRAF^V600E^* related modules

**Figure 2:**
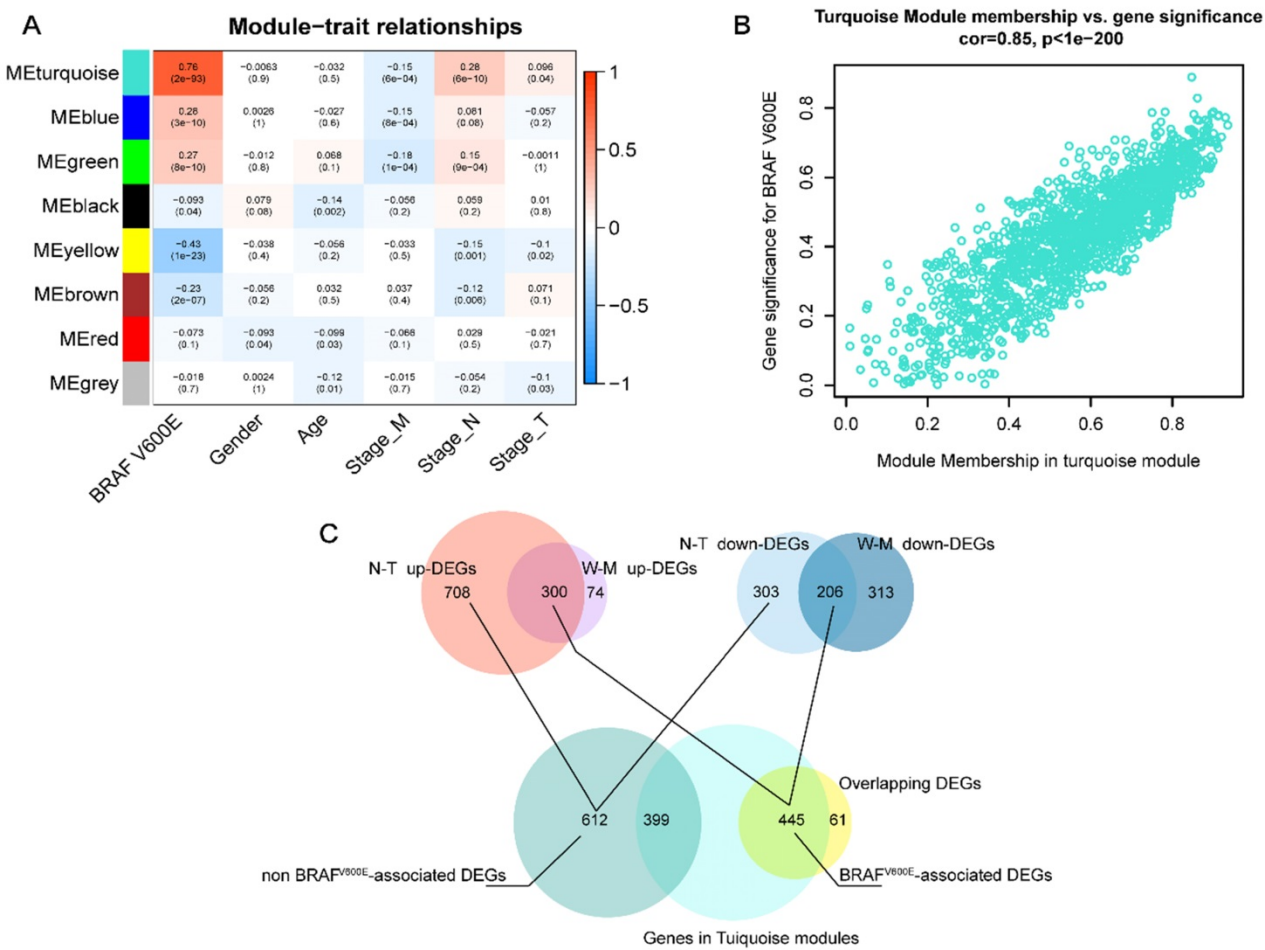
** Screening of *BRAF^V600E^*- associated genes. (A)** Result of weighted gene co-expression network analysis; Heatmap represents the modules‐trait relationship between module eigengene and clinical traits; The values in the heatmap represent correlation coefficients; parentheses, p values. ME: Module eigengene. **(B)** Scatter plots illustrating the correlation between gene significance (GS) and module membership (MM) of genes in module turquoise. The larger the value of MM, the more representative it is in the module. **(C)** Venn diagram showed the intersection of overlapping DEGs of N-T DEGs and M-W DEGs and genes in turquoise modules.

Next, we applied WGCNA to explore the co-expression network and find if there was any gene cluster highly related to the clinical traits that we are interested in. As shown in **figure 2A**, using WGCNA, we find that most modules were significantly correlated with *BRAF^V600E^* portrait, a total of 5 gene modules (*p*<0.01). Among them, the turquoise module had the strongest positive correlation with *BRAF^V600E^*. The correlation between the MM and GS of genes in the turquoise module were shown in **figure 2B**. Among the 1500 genes in the turquoise module, 445 genes overlapped with the DEGs specific to *BRAF^V600E^* were screened (**figure 2C**). These genes were defined as *BRAF^V600E^*-associated genes in PTC. In the non-*BRAF^V600E^*-specific DEGs, we filtered out 399 genes that overlapped with genes in the turquoise module, and the remaining 612 DEGs were defined as non-*BRAF^V600E^*-associated DEGs for PTCs.

### Function, pathway, and diseases enrichment analyses

**Figure 3:**
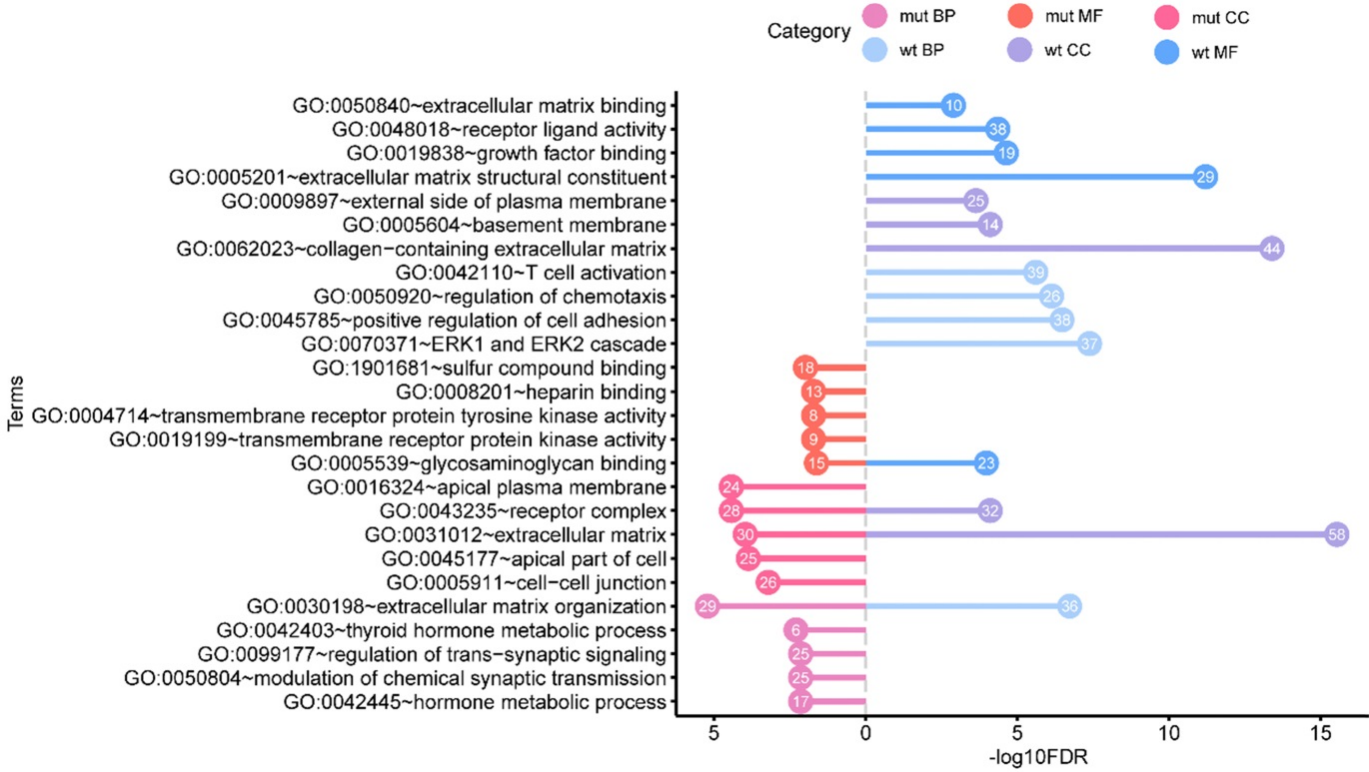
** Functional enrichment analysis of *BRAF^V600E^*- associated genes in comparing that of non *BRAF^V600E^*- associated genes. The** values in the bubble represent the gene count. mut: BRAF^V600E^ -associated genes; wt: non BRAF^V600E^ -associated genes; BP: biological process; CC: cellular component; MF: molecular function.

Aiming to better study the genetic differences in functions, the GO enrichment analysis, KEGG pathway enrichment analysis, and DO enrichment analysis of 445 *BRAF^V600E^*-associated DEGs and 612 non-*BRAF^V600E^*-associated DEGs were performed respectively. We obtained the enrichment results on GO and DO, and there was no significant KEGG pathway enriched. The top five most significant enrichment GO terms in each category were shown in **figure 3**.

The results of GO include terms of biological process (BP), cellular component (CC), and molecular function (MF). The most significant enrichment terms of BP, CC, MF for the *BRAF^V600E^*-associated DEGs were “extracellular matrix organization” (GO: 0030198, FDR = 5.97e-6), “receptor complex” (GO: 0043235, FDR = 3.66e-5) and “sulfur compound binding” (GO: 1901681, FDR = 0.01) respectively. The most significant enrichment terms for the non-*BRAF^V600E^*-associated DEGs were “ERK1 and ERK2 cascade” (GO: 0070371, FDR = 4.09e-8), “extracellular matrix” (GO: 0031012, FDR = 2.88e-16), and “extracellular matrix structural constituent” (GO: 1901681, FDR = 8.78e-15) respectively. In these GO terms, “extracellular matrix organization”, “receptor complex” and “extracellular matrix” were their common enrichment GO terms.

The top ten significant enrichment diseases for *BRAF^V600E^*-associated and non-associated DEGs diseases were shown in** table 3**. The most significant DO terms for them was “papillary thyroid carcinoma” (DOID: 3969, FDR=3e-4) and “retinal vascular disease” (DOID: 2462, FDR=3.87e-6) respectively.

### Targeted drug prediction for BRAF^V600E^-associated genes

To predict drugs for *BRAF^V600E^*-associated genes based on differential co-expression network, R package “DGCA” was applied to explore gene co-expression variations on the 445 BRAF^V600E^-associated genes. We obtained 22050 significant gene co-expression pairs at the absolute value of the correlation coefficients greater than 0.3 (*p*-value<0.05). Among these gene pairs, 750 gene pairs with opposite regulation in *BRAF^V600E^* and *BRAF^WT^* groups were defined as significantly differential co-expression pairs, involving 205 unique genes. Further, the 170 up-regulated genes and 35 down-regulated genes from the 205 genes were used to query the CREEDS database with their opposite signature from 875 manual single drug perturbations. The ranking of the predicted drugs was based on the Signed Jaccard Index, which represents signatures of reverse effects. The top 10 predicted drugs were shown in **table 4**. As is shown, the top predicted drug was Formoterol, an inhaled beta2-agonist used in the management of chronic obstructive pulmonary disease and asthma^21^, ^22^. Of note, the vemurafenib (also known as Plx4032), which is one of the most common BRAF^V600E^ inhibitors^23^, ^24^, appeared three times in the scope of the predicted drugs. The above results indicated that genes in the differential co-expression networks were quite associated with *BRAF^V600E^*, and might be the downstream target for BRAF^V600E^ inhibitor.

### Construction of co-expression network

**Figure 4:**
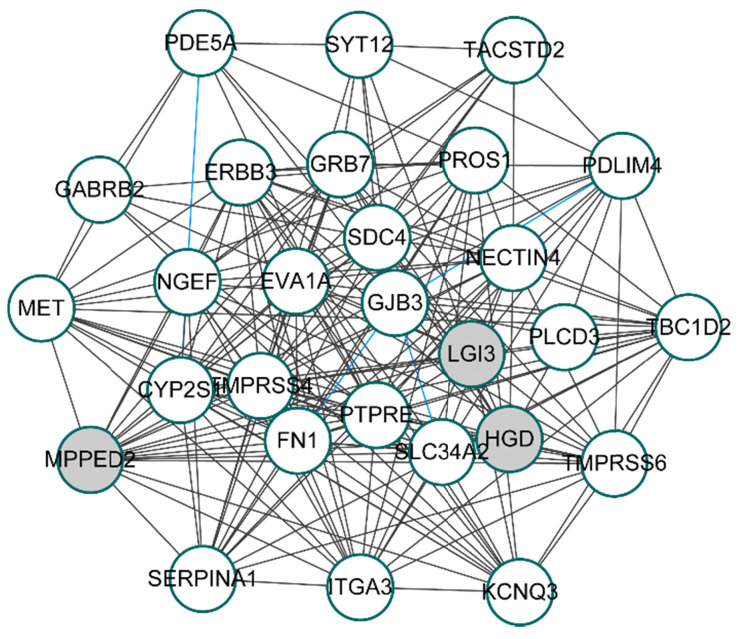
** Co-expression network of top 28 hub genes from *BRAF^V600E^*-associated genes.** White circles represent the up-regulated genes; Gray circles represent the down-regulated genes; Blue lines represent significant differential co-expression between *BRAF^V600E^*and *BRAF^WT^* group.

To unveil the genes that play potential key roles, 28 hub genes, whose absolute values of both MM and GS ranked in the top 50, were selected from turquoise modules for constructing the co-expression network. By calculating the correlation coefficients between 28 hub genes with R package “DGCA”, we got 174 significant gene pairs with correlation coefficients greater than 0.3 in both the *BRAF^V600E^* group and the *BRAF^WT^* group (*p*-value < 0.05). Based on the correlations among genes, the co-expression network of the 28 hub genes was shown in **figure 4**. Among the 174 gene pairs, three gene pairs had opposite correlations between the *BRAF^V600E^* and the *BRAF^WT^* groups.

### Identified key genes in the networks

**Figure 5:**
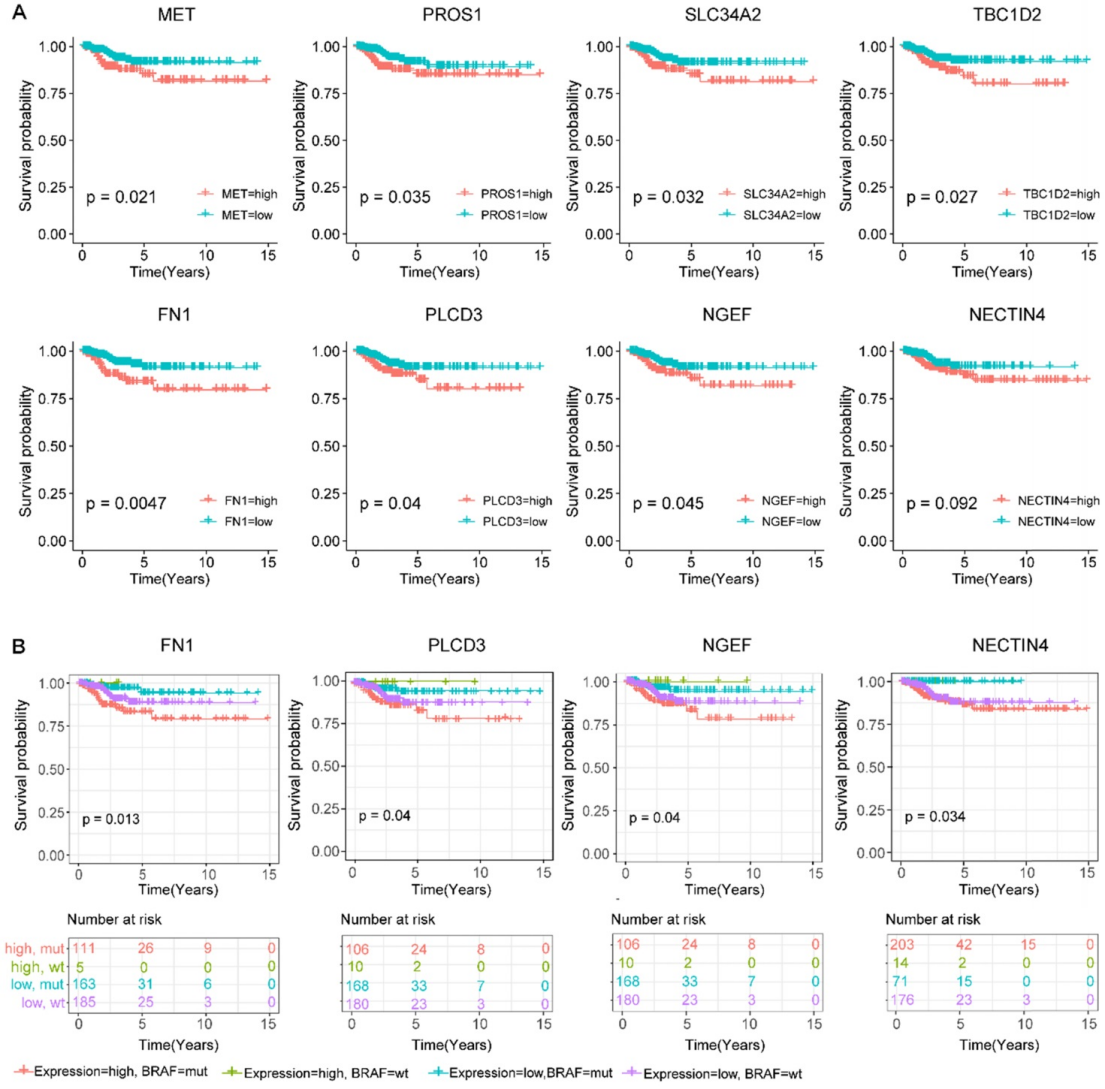
** Screening for *BRAF^V600E^*-associated prognostic biomarkers. (A)** Kaplan-Meier survival curves of eight prognostic biomarkers screened from hub genes. **(B)** Kaplan-Meier survival curves of prognostic biomarkers with the addictive effect of *BRAF^V600E^* mutation. High.mut: high expression of genes with *BRAF^V600E^* mutation; High.wt: high expression of genes without *BRAF^V600E^* mutation; Low.mut: low expression of genes with *BRAF^V600E^* mutation; Low.wt: low expression of genes without *BRAF^V600E^* mutation.

To predict the prognostic roles of genes in the co-expression network, relapse-free survival analyses were performed on genes in the above network. We also evaluated the relapse survival time of concomitant gene expression value and *BRAF* mutation status. Relapse-free survival (RFS) analyses of 464 PTC patients with full clinical information based on sequencing data revealed that higher expression levels of *MET*, *PROS1*, *SLC34A2*, *TBC1D2*, *FN1*, *PLCD3*, and *NGEF* were related to the shorter survival time of PTC patients (*p*-value < 0.05). Besides, cases with high expression of the *NECTIN4* also showed lower relapse-free probability though the result did not reach statistical significance (*p*-value = 0.092, **figure 5A**). These results indicate that these genes might function as oncogenes genes in the development of PTC.

To study the additive effect of *BRAF* mutations on the prognosis of these genes, we divided all the subjects into four groups based on the presence or absence of *BRAF^V600E^* mutations as well as the expression level of the above eight relapse-related genes, including the *BRAF^V600E^* high expression group, the *BRAF^V600E^* low expression group, the *BRAF^WT^* high expression group, and the *BRAF^WT^* low expression group. For four genes *FN1*, *PLCD3*, *NGEF*, and *NECTIN4*, the *BRAF^V600E^* high expression group was prone to relapse than the other groups (**figure 5B**). Of note, the expression level of the *NECTIN4* alone though effected on the relapse on PTC without significance, patients with coexistence of the overexpressed *NECTIN4* and the *BRAF^V600E^* mutation had shorter relapse-free survival time.

**Figure 6:**
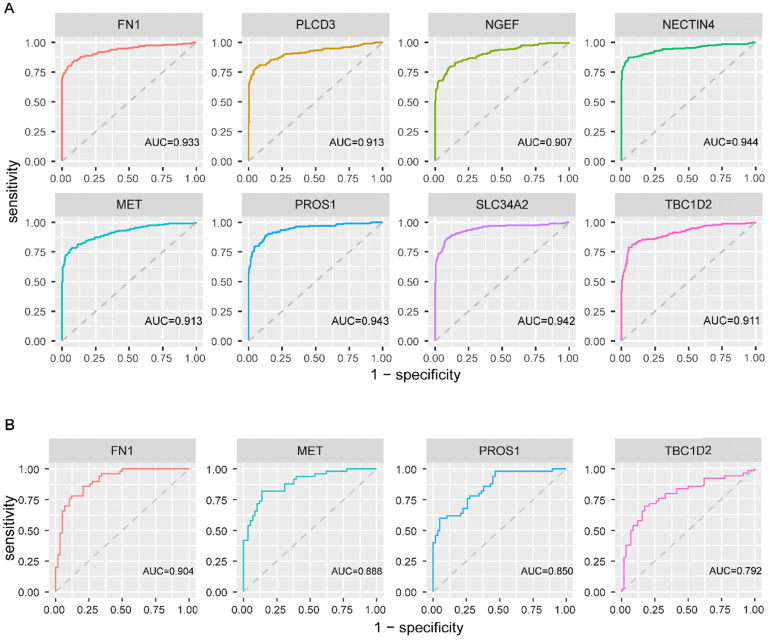
** Receiver operating characteristic curve analysis and validation of screened biomarkers. (A)** Receiver operating characteristic curve analysis of 8 screened biomarkers in TCGA. **(B)** Receiver operating characteristic curve analysis of 4 biomarkers existed in GEO. AUC: area under the curve

To explore the diagnostic values of the above eight genes, ROC curve analysis was conducted on 482 PTC patients from TCGA. The results revealed that all the eight genes had a good performance in distinguishing PTC patients with *BRAF^V600E^* PTC patients from *BRAF^WT^* PTC patients, with the AUC exceeded 0.9 (**figure 6A**). Further, this finding was validated based on 108 PTC cases from three GEO datasets. *FN1*, *MET*, *PROS1*, and *TBC1D2* were detected in GEO datasets. Same as in TCGA, the expression value of these 4 genes was higher in the *BRAF^V600E^* group than that in the *BRAF^WT^* group. The AUC of the ROC analyses for these four genes exceeded 0.7 (**figure 6B**), indicating their certain diagnostic value for *BRAF^V600E^* PTCs in GEO.

### Verification of the differential expression levels of biomarkers at different groups

**Figure 7:**
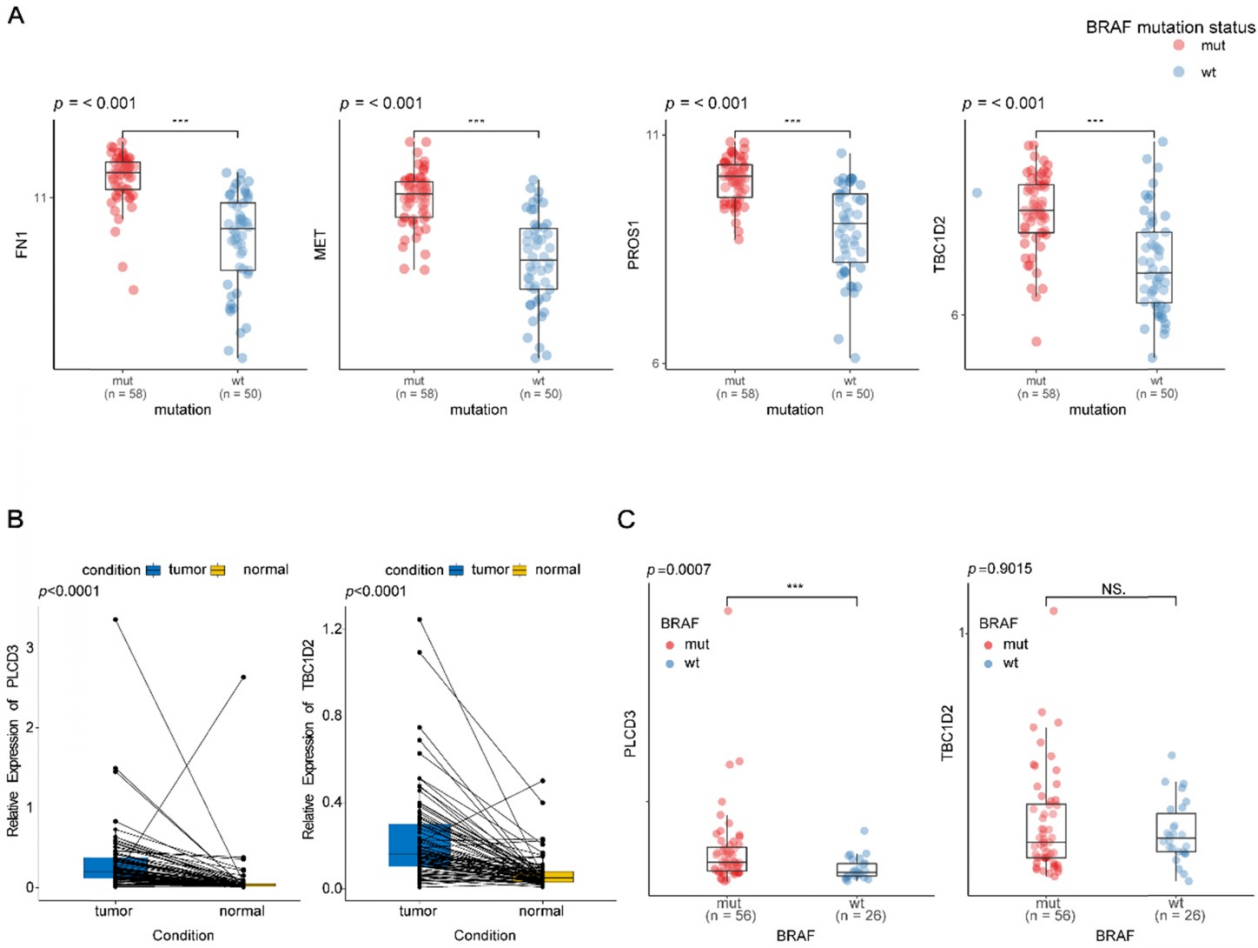
** Validation of the different expression levels of the biomarkers at different groups. (A)** Box plot of *FN1*, *MET*,* PROS1* and *TBC1D2* expression at different *BRAF* mutation status in papillary thyroid carcinoma existed in the GEO database. Y-axis represents the log2 transformation of RNAseq-HT seq FPKM‐UQ+1. FPKM‐UQ: upper quartile FPKM, a modified FPKM calculation (FPKM: fragments perper Kilobase of transcript perper Million mapped reads); HTseq: high‐throughput sequencing; RNAseq: RNA sequencing. **(B)** qPCR validation of two novel biomarkers expression levels between matched normal and tumor tissues from patients with papillary thyroid carcinoma. **(C)** qPCR validation of two novel biomarkers expression levels at different *BRAF* mutation status in papillary thyroid carcinoma. * * *:p < .001; NS: No Significance.

To validate the differential expression levels of the biomarkers we screened, their expression patterns were further analyzed in the GEO database. Though there were only 4 of them, including *FN1*,* MET*,* PROS1*, and *TBC1D2*, were detected in the GEO database, their expression patterns at different *BRAF^V600E^* status in GEO were in accordance with that in TCGA database (**Figure 7A**).

As the expression patterns of *TBC1D2* and *PLCD3* in PTC have not been reported in existing studies, qRT-PCR was used to validate the expressions of two novel recurrence-associated biomarkers in our hub co-expression network from WGCNA (*TBC1D2* and *PLCD3*). The results in **figure 7B** showed that there were significant differences for two genes between normal tissues and tumor tissues from PTC patients (*p*<0.0001). *PLCD3* was significantly overexpressed in the *BRAF^V600E^* PTC patients compared with BRAF^WT^ patients (*p*=0.0007). However, no statistical significance was found for the differential expression of *TBC1D2* between *BRAF^WT^* and *BRAF^V600E^* patients *(p*=0.9015). Whereas both the two genes appeared a high expression tendency in the *BRAF^V600E^* patients (**Figure 7C**).

## Discussion

In this study, we screened* BRAF^V600E^*-associated genes in PTC based on differentially expressed gene analyses and WGCNA. Comprehensive analyses were performed through functional enrichment, targeted drug prediction, and hub genes screening. Finally, we identified eight *BRAF^V600E^***-**associated key genes related to the relapse of PTC from the co-expression network based on hub genes. ROC analysis conformed eight genes could serve as biomarker candidates to distinguish *BRAF^V600E^* PTC from *BRAF^WT^* PTC. Based on the findings for prognosis and diagnosis and the prediction of targeted drugs, we obtained several potential targeted drugs that could be used in combination with BRAFi in the treatment of *BRAF^V600E^* PTC to overcome their resistance to BRAFi.

Functional enrichment analysis showed that although *BRAF^V600E^***-**associated genes and non-*BRAF^V600E^***-**associated genes were enriched in several common functions, their differences are more significant. It was noteworthy that "ERK1 and ERK2 cascade" was the most significantly enriched GO terms of non-*BRAF^V600E^***-**associated genes but not the *BRAF^V600E^***-**associated genes, corresponding with the fact that *BRAF^V600E^* mutation caused abnormal regulation of MAPK and its downstream EKR signaling pathway^25^. For MF, the enrichment result indicated *BRAF^V600E^***-**associated genes were not only enriched in binding to sulfide and heparin but also played a certain role in the activity of receptor kinases and tyrosine kinases, which has been confirmed in a variety of *BRAF* mutant tumors, including thyroid cancer, colorectal cancer, melanoma, etc^26^-^30^. For CC, compared with the result of functional enrichment of non-*BRAF^V600E^*-associated genes, *BRAF^V600E^*-associated genes were mainly enriched in the “apical part of cell”, “apical plasma of cell,” and “cell-cell junction”, indicating that *BRAF* mutations might play roles in the connection and interaction of tumor cells. Enrichment in BP function indicated that *BRAF* mutations may cause changes in the synthesis and metabolism of hormones and compounds which were related to the thyroid gland. The results of DO enrichment analyses suggested that *BRAF^V600E^*-associated genes were enriched in “papillary thyroid carcinoma”, while non- *BRAF^V600E^*-associated genes were enriched in “retinal vascular disease” respectively. This result indicated that *BRAF^V600E^*-associated genes we screened with WGCNA based on DEGs were more related to PTC.

Among the top ten drugs predicted by genes based on differential co-expressed network, vemurafenib is a classical BRAFi applied in a variety of cancers with *BRAF^V600E^* mutation, including PTC 27, 31, 32. This finding further illustrated there was a certain correlation between the *BRAF^V600E^* mutation and the genes we identified. Carboplatin, an antineoplastic agent, was used to treat various forms of cancer, especially advanced ovarian carcinoma^33^. It has been reported that a *BRAF^V600E^* PTC patient with tall cell variant and squamous transformation responded well to treatment with concurrent chemotherapy with carboplatin and paclitaxel along with radiation^34^. Carboplatin/paclitaxel could also be used in the chemotherapy of anaplastic thyroid cancer (ATC)^35^. The combination of vemurafenib, carboplatin, and paclitaxel demonstrated certain tolerance and activity in patients with advanced melanoma and *BRAF^V600E^* mutations^36^. Therefore, carboplatin/ paclitaxel combining with BRAFi may also be a feasible treatment strategy for advanced PTC and ATC, especially those with *BRAF^V600E^* mutation. Other drugs predicted by genes in the differential co-expression network seemed to be unrelated to thyroid cancer.

For the eight key genes screened in this study, although only four of them reappeared in the GEO database, their expression patterns and diagnostic value were consistent with our analysis in TCGA, indicating the reliability of our result. *MET* and *FN1* were the two genes that have been confirmed as oncogenes associated with *BRAF* mutations in PTC. In PTC and melanoma, the upregulation of *MET* and *ERBB3* expression level induced by BRAFi conferred BRAFi resistance to *BRAF^V600E^* mutant carcinomas, and inhibitors of MET and ERBB family could reserve the resistance to BRAFi effectively^37^-^41^. *ERBB3* was also a hub gene in our hub co-expression network. The efficacy of MET inhibitor combining ERBB family inhibitor has been validated in cutaneous malignant melanoma^42^. Combining with our analysis, these existed researches suggesting the combination of MET and ERBB inhibitors was a potential therapeutic strategy for PTC with resistance to BRAFi. In accordance with our findings, *FN1* was a well-known prognostic biomarker associated with *BRAF^V600E^* mutation and the recurrence of thyroid cancer^43^, ^44^. Multiple studies have shown that high expression of *FN1* was related to aggressiveness and *BRAF^V600E^* mutation in PTC 45, 46.

For the rest six genes, *SLC34A2*, *PROS1*, *NACTIN4*, and *NGEF* were the genes that had been confirmed to be up-regulated in PTC, but there was no direct evidence for their association with *BRAF^V600E^* mutation. *SLC34A2* was an oncogene highly expressed in a variety of tumors, including lung, ovarian, colorectal, and thyroid cancer, and was associated with tumor metastasis and recurrence of colorectal and thyroid cancer^47^-^50^. *In vivo*, experiments showed that *SLC34A2* inhibition combining with MK2206 (an allosteric AKT1/2 inhibitor), exhibited significant antitumorigenic potential^48^. Anti-NaPi2b antibody-drug conjugate (ADC), a targeted ADC for *SLC34A2*, was also proven to be effective and safe in non-small cell lung cancer and ovarian cancer^51^. Although the effectiveness of this drug for PTC has not been explored yet, we associated it with *BRAF* mutations for the first time, providing a theoretical basis for the combination therapy of *SLC34A2* inhibition and BRAFi. The underlying functions of *PROS1* and *NGEF* in PTC need further exploration. As one of the ligands for the TAM (Tyro3, Axl, and Mertk) receptor tyrosine kinases, *PROS1* was proven to be a strong stimulator of p-Erk through ProS1-Tyro3-Erk signaling pathway in some human cancer cell lines^52^, which indicated *PROS1* may also participate in the ERK pathway downstream of *BRAF^V600E^*. *NATIN4* was reported to be associated with lymph node metastasis and function as a promoter in the colony formation, proliferation, migration, and invasion of PTC cell lines^53^.* TBC1D2* and *PLCD3* were two novel potential biomarkers without previous reports for PTCs. Nevertheless, our qRT-PCR experiments confirmed their high expression in PTC tumor tissues compared to normal tissues. The experimental results did not indicate a statistical difference in the expression of *TBC1D2* between the *BRAF^V600E^* PTC patients and *BRAF^WT^* PTC patients possible due to the fact that our sample size was insufficient to reflect statistical significance. Whereas the tendency of the *TBC1D2* and *PLCD3* expression levels in *BRAF^V600E^* PTC patients and *BRAF^WT^* PTC patients were consistent with our bioinformatics analysis results, that is, the expression value of both the two genes were higher in *BRAF^V600E^* PTC patients. However, the function and the relationship with *BRAF^V600E^* of these genes need further experimental investigation.

In conclusion, through a comprehensive analysis of *BRAF^V600E^***-**associated genes and their hub co-expression networks, we had further understood the functional differences between the *BRAF^V600E^***-** associated genes and the non-*BRAF^V600E^***-**associated genes. Eight *BRAF^V600E^* -associated biomarkers with both prognostic and diagnostic values were identified, and six of them were the novel findings related to *BRAF^V600E^* mutation in PTC. Based on the literature review of these biomarker candidates and the prediction of targeted drugs for genes from a differentially co-expressed network, we obtained four potential drugs that could be used in combination with BRAFi for PTC therapy, including MET inhibitor, ERBB3 inhibitor, anti-NaPi2b ADC, and carboplatin. Our findings had a certain theoretical value for accurate personalized treatment of PTC to obtain a better harm-benefit balance and provided new ideas for overcoming the resistance to BRAFi.

However, there are still some limitations to our study. Since the expression data from GEO were expression profiling by array, some genes in TCGA could not be found in GEO expression profiling, only four of our key genes have been verified. Limited by our sample scale, the difference in the expression of the two novel genes between the *BRAF^V600E^* and *BRAF^WT^* groups has not been fully experimentally confirmed. Besides, for biomarkers with unclear function and molecular mechanisms, our bioinformatics analysis only played a predictive role, and their function and potential as a drug target need to be validated by further experimental and clinical research.

## Figures and Tables

**Table 1:** Clinical information of 482 PTC cases in WGCNA

	BRAF^V600E^ (n=283)	BRAF^WT^(n=199)	Overall (n=482)	*p*-value
**Age**				
Median [Min, Max]	47.0 [15.0, 89.0]	46.0 [15.0, 88.0]	46.0 [15.0, 89.0]	0.518
**Vital status, n (%)**				1
Alive	274 (96.8%)	194 (97.5%)	468 (97.1%)	
Dead	8 (2.8%)	5 (2.5%)	13 (2.7%)	
Missing	1 (0.4%)	0 (0%)	1 (0.2%)	
**Extrathyroid extension status, n (%)**				<0.001
None	162 (57.2%)	157 (78.9%)	319 (66.2%)	
Minimal (T3)	100 (35.3%)	31 (15.6%)	131 (27.2%)	
Moderate/Advanced (T4a)	16 (5.7%)	1 (0.5%)	17 (3.5%)	
Very Advanced (T4b)	0 (0%)	1 (0.5%)	1 (0.2%)	
Missing	5 (1.8%)	9 (4.5%)	14 (2.9%)	
**Gender, n (%)**				0.845
Female	207 (73.1%)	148 (74.4%)	355 (73.7%)	
Male	76 (26.9%)	51 (25.6%)	127 (26.3%)	
**Histological type, n (%)**				<0.001
Other, specify	4 (1.4%)	5 (2.5%)	9 (1.9%)	
Classical/usual	233 (82.3%)	106 (53.3%)	339 (70.3%)	
Follicular (>= 99% follicular patterned)	13 (4.6%)	86 (43.2%)	99 (20.5%)	
Tall Cell (>= 50% tall cell features)	33 (11.7%)	2 (1.0%)	35 (7.3%)	
**Pathologic M, n (%)**				0.026
M0	175 (61.8%)	98 (49.2%)	273 (56.6%)	
M1	5 (1.8%)	4 (2.0%)	9 (1.9%)	
MX	103 (36.4%)	96 (48.2%)	199 (41.3%)	
Missing	0 (0%)	1 (0.5%)	1 (0.2%)	
**Pathologic N, n (%)**				<0.001
N0	106 (37.5%)	113 (56.8%)	219 (45.4%)	
N1	35 (12.4%)	21 (10.6%)	56 (11.6%)	
N1a	70 (24.7%)	15 (7.5%)	85 (17.6%)	
N1b	51 (18.0%)	22 (11.1%)	73 (15.1%)	
NX	21 (7.4%)	28 (14.1%)	49 (10.2%)	
**Pathologic T, n (%)**				0.001
T1	22 (7.8%)	19 (9.5%)	41 (8.5%)	
T1a	9 (3.2%)	9 (4.5%)	18 (3.7%)	
T1b	44 (15.5%)	34 (17.1%)	78 (16.2%)	
T2	78 (27.6%)	80 (40.2%)	158 (32.8%)	
T3	112 (39.6%)	52 (26.1%)	164 (34.0%)	
T4	5 (1.8%)	4 (2.0%)	9 (1.9%)	
T4a	13 (4.6%)	0 (0%)	13 (2.7%)	
TX	0 (0%)	1 (0.5%)	1 (0.2%)	
**Pathologic stage, n (%)**				<0.001
Stage I	150 (53.0%)	118 (59.3%)	268 (55.6%)	
Stage II	18 (6.4%)	33 (16.6%)	51 (10.6%)	
Stage III	75 (26.5%)	33 (16.6%)	108 (22.4%)	
Stage IV	0 (0%)	2 (1.0%)	2 (0.4%)	
Stage IVA	35 (12.4%)	10 (5.0%)	45 (9.3%)	
Stage IVC	4 (1.4%)	2 (1.0%)	6 (1.2%)	
Missing	1 (0.4%)	1 (0.5%)	2 (0.4%)	

**Table 2:** The list of the primers for sequencing and qPCR

Genes	Primer name	Primer sequence (from 5' to 3')
BRAF	BRAF-F	TCATAATGCTTGCTCTGATAGGA
	BRAF-R	GGCCAAAAATTTAATCAGTGGA
PLCD3	PLCD3-F	GGGCTGCGGATGAACTCAG
	PLCD3-R	CACTGCCCATTGACTAGGAAG
TBC1D2	TBC1D2-F	ACAACATCCGTGGCAACAAG
	TBC1D2-R	CTTTCTGAGCGAAACTGATGGT
GAPDH	h-GAPDH-F	TCTCTGCTCCTCCTGTTCGA
	h-GAPDH-R	GCGCCCAATACGACCAAATC

**Table 3:** Top 10 results of Disease Ontology enrichment analysis for BRAF^V600E^ -associated genes and non BRAF^V600E^-associated genes

Terms	Counts	pValue	FDR	Gene Type
DOID:3969~papillary thyroid carcinoma	16	5.17E-07	0.0003	BRAF^V600E^ -associated
DOID:170~endocrine gland cancer	39	1.14E-06	0.0004	BRAF^V600E^ -associated
DOID:3963~thyroid carcinoma	21	4.13E-06	0.0009	BRAF^V600E^ -associated
DOID:1781~thyroid cancer	21	1.07E-05	0.0017	BRAF^V600E^ -associated
DOID:3113~papillary carcinoma	8	1.49E-04	0.0194	BRAF^V600E^ -associated
DOID:10124~corneal disease	9	2.55E-04	0.0278	BRAF^V600E^ -associated
DOID:50~thyroid gland disease	15	3.29E-04	0.0307	BRAF^V600E^ -associated
DOID:28~endocrine system disease	27	4.22E-04	0.0341	BRAF^V600E^ -associated
DOID:4074~pancreas adenocarcinoma	15	4.96E-04	0.0341	BRAF^V600E^ -associated
DOID:1793~pancreatic cancer	23	5.88E-04	0.0341	BRAF^V600E^ -associated
DOID:2462~retinal vascular disease	12	3.87E-06	0.0014	non BRAF^V600E^- associated
DOID:8947~diabetic retinopathy	12	3.87E-06	0.0014	non BRAF^V600E^ -associated
DOID:263~kidney cancer	38	1.06E-05	0.0021	non BRAF^V600E^ -associated
DOID:13207~proliferative diabetic retinopathy	9	1.14E-05	0.0021	non BRAF^V600E^ -associated
DOID:4451~renal carcinoma	34	1.70E-05	0.0023	non BRAF^V600E^ -associated
DOID:3996~urinary system cancer	41	1.90E-05	0.0023	non BRAF^V600E^ -associated
DOID:1036~chronic leukemia	23	2.36E-05	0.0024	non BRAF^V600E^ -associated
DOID:4450~renal cell carcinoma	31	3.16E-05	0.0024	non BRAF^V600E^ -associated
DOID:1107~esophageal carcinoma	16	3.48E-05	0.0024	non BRAF^V600E^ -associated
DOID:18~urinary system disease	39	3.52E-05	0.0024	non BRAF^V600E^ -associated

**Table 4:** Top 10 predicted drugs for BRAF^V600E^-associated genes

ID	Name	GEO ID	Signed Jaccard Index
drug:2631	Formoterol	GSE30242	-0.01279
drug:3204	Tibolone	GSE12446	-0.0124
drug:2569	Plx4032	GSE24862	-0.01231
drug:3200	Hypochlorous acid	GSE11630	-0.0109
drug:3176	Carboplatin	GSE49577	-0.01016
drug:2565	Plx4032	GSE24862	-0.00985
drug:3202	Hypochlorous acid	GSE11630	-0.0096
drug:3354	Sodium arsenite	GSE11056	-0.00945
drug:2509	Vemurafenib	GSE63790	-0.00944

## Abbreviations

PTC: papillary thyroid cancer
BRAF: B-type Raf kinase
BRAFV600E: B-type Raf kinase V600E mutation
BRAFi: BRAF inhibitors
BRAFWT: BRAF wild-type
TCGA: The Cancer Genome Atlas
CREEDS: crowd extracted expression of differential signatures
ROC: receiver operating characteristic
WGCNA: weighted gene co-expression network analysis
GEO: Gene Expression Omnibus
CPM: counts per million
DEG: differentially expressed gene
N-T DEGs: DEGs between normal tissues and tumor tissues
WT-V600E DEGs: DEGs between BRAF wide type and BRAFV600E tumor tissues
MM: module membership
GS: Gene Significance
GO: Gene Ontology
KEGG: Kyoto Encyclopedia of Genes and Genomes
DO: Disease Ontology
FDR: false discovery rate
BP: biological process
CC: cellular component
MF: molecular function
RFS: relapse-free survival
ATC: anaplastic thyroid cancer
